# Fight or Flight Freeze- A Case Report Paper, PTSD patient

**DOI:** 10.1192/j.eurpsy.2025.1947

**Published:** 2025-08-26

**Authors:** A. Botica, N. Kurtovic, M. Strikic, T. Glavina

**Affiliations:** 1 Psychiatry, University Hospital Centre; 2 Psychiatry, 2Mental Health Service, Teaching Institute for Public Health, Split, Croatia

## Abstract

**Introduction:**

Post-traumatic stress disorder (PTSD) is a mental health condition that violates everyday functioning, and it is a result of a traumatic event or life-threatening event. Symptoms of PTSD are nightmares, flashbacks, symptoms of negative emotions and perception, avoidance behavior, and social avoidance and it can affect children. Dissociative symptoms are a part of dissociative disorder in the form of anxiety disorder, but these symptoms can occur in dissociative form of PTSD.

**Objectives:**

The goal of this paper is to present a patient who is under neurological treatment, diagnosed with spastic paraplegia, and under psychiatric treatment diagnosed with PTSD, depression, and possible dissociative disorder.

**Methods:**

This is a case report paper. A male patient, 57 years old, participated in Homeland War in Croatia for a duration of 18 months. He experienced many traumatic events and life-threatening events, such as the death of his friends. The first psychiatric symptoms occurred after war experiences, and the symptoms manifested as headache, insomnia, grumpiness, and paralyzing fear which led to the feeling that his legs were “taken away”. Similar symptoms persisted even after the war ended and in other stressful situations along with difficulty walking. He went under neurological testing and it turns out that it might be spastic paraplegia. He was diagnosed with prolactinoma, and since 2019 he has been in psychiatric ambulance treatment in the Daily Hospital of Clinical Hospital Split, diagnosed with PTSD, depression, and dissociative disorder.

**Results:**

We used psychotherapy methods such as socio-therapeutic and group psychotherapy, along with pharmacology therapy (sertraline, diazepam, olanzapine).

**Conclusions:**

Pharmacology interventions might affect PTSD patients in a positive way and help them to function in everyday activities.

**Disclosure of Interest:**

None Declared

